# Discrimination of Second Language Vowel Contrasts and the Role of Phonological Short-Term Memory and Nonverbal Intelligence

**DOI:** 10.1007/s10936-024-10038-z

**Published:** 2024-02-04

**Authors:** Georgios P. Georgiou, Aretousa Giannakou

**Affiliations:** 1 Department of Languages and Literature, University of Nicosia, Nicosia, Cyprus; 2 Phonetic Lab, University of Nicosia, Nicosia, Cyprus

**Keywords:** Speech perception, Phonological short-term memory, Nonverbal intelligence, Second language, Greek, English

## Abstract

Although extensive research has focused on the perceptual abilities of second language (L2) learners, a significant gap persists in understanding how cognitive functions like phonological short-term memory (PSTM) and nonverbal intelligence (IQ) impact L2 speech perception. This study sets out to investigate the discrimination of L2 English monophthongal vowel contrasts and to assess the effect of PSTM and nonverbal IQ on L2 speech perception. The participants consisted of adult monolingually-raised Greek speakers, who completed an AX discrimination test, a digit span test, and a nonverbal intelligence test. A control group of English speakers also completed the AX test. Data were analyzed using Bayesian regression models. The results revealed that Greek speakers exhibited below chance discrimination for the majority of L2 vowel contrasts, consistently underperforming in comparison to the control group. Intriguingly, the study did not provide substantial evidence in favor of more accurate discrimination of L2 contrasts by Greek participants with high PSTM compared to those with low PSTM. However, the study yielded compelling evidence indicating that Greek participants with higher IQ demonstrated superior accuracy in discriminating most L2 contrasts compared to their lower IQ counterparts. The limited influence of PSTM on speech perception suggests the need for further exploration, considering the potential impact of test methodologies and the intricate interplay of other confounding factors. Furthermore, the study uncovers a noteworthy relationship between nonverbal IQ and L2 speech perception, likely linked with the association of high IQ with enhanced attentional capacities, information processing abilities, and learning skills—all of which are pivotal for accurate speech perception.

## Introduction

The difficulties observed in adults in accurately distinguishing second language (L2) sound contrasts is mainly attributed to the strong effect of their first language (L1) (Evans & Alshangiti, 2018; Georgiou, 2022a, 2024; Iverson et al., 2003; Shinohara & Iverson, 2018). Considering that L2 sounds are mapped in terms of L1 categories in the human mind (e.g., see Best & Tyler, 2007; Cebrian, 2006; Georgiou et al., 2020a, 2020b; Mayr & Escudero, 2010; Schmidt, 2018), L2 sounds mapped to a single L1 category are particularly difficult to distinguish. For example, Georgiou et al. (2020b) found that Russian learners of English struggled to discriminate the English /æ/–/e/contrast since they assimilated both English vowels to the same Russian category, that is, /e/. This single-category mapping might be fostered by the size and complexity of the L1-L2 systems. For instance, Iverson and Evans (2007) reported that speakers of L1s with large and more complex vowel systems (German and Norwegian) identified the L2 English vowels more accurately than speakers of L1s with smaller and less complex vowel systems (Spanish and French). Nevertheless, more recent studies argued that having a large and more complex vowel system does not guarantee a discrimination advantage. Elvin et al. (2014) found that Australian English listeners do not discriminate Brazilian Portuguese vowel contrasts better than Spanish listeners despite the large size and complexity of the Australian English vowel system and the small size and simplicity of the Spanish vowel system. The authors concluded that crosslinguistic properties but not crosslinguistic vowel systems predicted the difficulty of learners in discriminating the L2 contrasts. The size and complexity of L1 and L2 systems can be considered together with the acoustic similarity between L1-L2 sounds to predict the speakers’ L2 perception patterns (see Alispahic et al., 2017; Georgiou, 2023d).

Discrimination predictions are usually based on the theoretical account of specific speech models. A recent addition is the Universal Perceptual Model (UPM) (Georgiou & Dimitriou, 2023; Georgiou, 2021a, 2023c), which accounts for the difficulties of learners in the discrimination of L2 sound contrasts. Being consistent with other models, UPM also suggests that the acoustic characteristics of L1-L2 sounds may be useful for the assessment of speakers’ discrimination abilities over the L2 contrasts. More specifically, it proposes that *perceptual overlapping* between two contrastive L2 sounds against an L1 sound can predict the discrimination difficulty of the L2 pair. In this context, two L2 contrasts can be *completely overlapping*, *partially overlapping* and *nonoverlapping*. Completely overlapping contrasts are expected to be discriminated worse than partially overlapping contrasts, and the latter are expected to be discriminated worse than nonoverlapping contrasts.

Apart from the linguistic account, the contribution of several factors such as age of learning (Flege et al., 1995), length of residence (Meador et al., 2000), L1-L2 use (Flege et al., 1997), quality and quantity of L2 input (Flege & Liu, 2001), gender (Flege & Fletcher, 1992), etc. has been investigated in a large number of studies. The role of cognitive functions such as phonological short-term memory (PSTM) and intelligence (IQ) has received less scientific attention. PSTM constitutes an integral component of the multidimensional working memory (WM) system, alongside dimensions associated with attentional resource capacity and allocation, as well as processing speed (Montgomery et al., 2009). Baddeley et al. (1984) reported that the PSTM model contains two main elements: a phonological buffer or store designed for the temporary retention of memory traces lasting a few seconds, and a subvocal rehearsal process employed for the purpose of rejuvenating these memory traces. General intelligence, or the so-called *g* factor, originally proposed by Spearman (1904), comprises a psychometric construction of cognitive abilities and human intelligence. Cattell (1963) refers to two types of general intelligence, fluid and crystallized. Fluid intelligence shows the ability of individuals to solve problems and reason and is often associated with nonverbal skills. Crystallized intelligence emerges from past experience and is improved over time as individuals increase their knowledge; this knowledge is language- and culture-specific (Dryden et al., 2017). This type of intelligence is based on verbal skills.

Previous work that attempted to define the relationship between PSTM and language studied the effect of PSTM on L2 oral fluency (O’Brien et al., 2006), vocabulary learning (French & O’Brien, 2008; Martin & Ellis, 2012), and grammar aspects (Masoura & Gatherole, 2005). There is less evidence regarding the interface between PSTM and L2 speech perception. A relationship between PSTM and speech perception patterns is expected since PSTM enables learners to temporarily store nonnative sounds, facilitating the ability to perceive and differentiate them from the familiar sounds of their L1. By employing the rehearsal mechanism, nonnative sounds can effectively be reinforced, thus aiding in the development of enduring and robust mental representations. This, in turn, enhances the capacity for long-term recognition and differentiation of these sounds. MacKay et al. (2001) delved into the identification of English vowels by Italian speakers. Their research unveiled that PSTM significantly predicted the identification of word-final English consonants, accounting for approximately 15% of the variance, as well as the identification of word-initial English consonants, where it explained about 8% of the variance. These findings were deemed noteworthy for understanding and predicting the identification patterns exhibited by the speakers. Lengeris and Nicolaides (2014) examined the identification of English consonants by Greek listeners in quiet and noise contexts and the effect of PSTM on this identification using a nonword repetition task. The findings showed that PSTM highly correlated with the capacity of the listeners to identify English consonants. Aliaga-Garcia et al. (2011) indicated that Catalan-Spanish learners of English with higher PSTM capacities had higher accuracy scores and perceptual gains from phonetic training compared to Catalan-Spanish learners of English with lower PSTM capacities, highlighting the significant role of PSTM in L2 vowel discrimination. Along the same lines, Safronova (2016) found a positive correlation between PSTM and discrimination performance over L2 English contrasts by Spanish-Catalan learners of English. In addition, larger PSTM capacity was associated with better performance in the distinction of these contrasts. However, this result could not be verified for Azerbaijani learners of English (Ghaffarvand Mokari & Werner, 2019). The authors trained the learners in the discrimination of L2 English contrasts, concluding that there was no correlation between gains from high variability phonetic training and PSTM—thus, the role of PSTM in phonological learning was insignificant. Similarly, Safronova and Mora (2012) indicated no advantage for Catalan learners of English with a large PSTM capacity in more accurately identifying the English /iː/–/ɪ/ contrast in comparison to learners with a low PSTM capacity. PSTM is measured using a variety of methods including nonword repetition (Lengeris & Nicolaides, 2014) or serial nonword recognition tasks (SNWR) (Cerviño-Povedano & Mora, 2010; Ghaffarvand Mokari & Werner, 2019), immediate serial recall (Tree & Playfoot, 2019) and digit span tests (Kim et al., 2016) among other. Although the list of studies is not exhaustive, previous findings suggest mixed results regarding the effect of PSTM on the learners’ speech perception abilities.

There is evidence focusing on the link between L2 learning and IQ. In some studies, high- vs low-achieving L2 learners were found to differ from each other in terms of verbal/nonverbal IQ (e.g., Sparks et al., 1992), while some other studies indicated no such difference (e.g., Sparks et al., 2012). In an earlier study, Carroll (1962) suggested that while L2 aptitude does not depend on IQ, there might be an association between general IQ and the capability of cognitive mechanisms in supporting L2 proficiency. Łockiewicz et al. (2018) explored the predictors of foreign language learning by L1 Polish preschooler learners of English as a foreign language. The authors observed that nonverbal IQ was a good predictor of the oral English skills of children. Woumans et al. (2019) examined the relationship between L2 acquisition and nonverbal cognitive abilities, identifying nonverbal IQ as one of the contributing factors to the acquisition of L2 Dutch vocabulary, as higher performance on Dutch L2 vocabulary correlated with higher nonverbal IQ progress. A similar positive relationship between nonverbal IQ and L2 vocabulary development was observed by Daller and Ongun (2018) in Turkish-English successive bilingual children. Research regarding the effect of IQ on phonetic aspects is scarce. Rota and Reiterer (2009) examined among other the correlation between verbal and nonverbal IQ and phonetic abilities. Their results suggested no correlation between verbal and nonverbal IQ and pronunciation, while a correlation was found between verbal IQ and phonetic coding ability. Similarly, Christiner et al. (2018) reported that nonverbal IQ did not correlate with language phonetic aptitude. In contrast, one recent study demonstrated that Cypriot Greek speakers of L2 English with a high nonverbal IQ capacity discriminated the majority of L2 vowel contrasts more accurately than the corresponding speakers with a low nonverbal IQ capacity (Georgiou, 2023a). Some of the most popular measures of nonverbal IQ are the Wechsler Abbreviated Scale of Intelligence (WASI) (Wechsler, 1999), the Wechsler Adult Intelligence Scale (WAIS) (Wechsler, 2008), and the Raven Progressive Matrices tests (Raven et al., 2000). All in all, the very few studies in the literature manifest contradictory findings about the effect of nonverbal IQ on L2 speech perception.

This paper aims to answer two main questions: (i) how do Greek speakers of L2 English discriminate specific pairs of English monophthongal vowel contrasts (/iː/–/ɪ/, /iː/–/e/, /ɑː/–/ʌ/, /æ/–/ɑː/, /ɔː/–/ɒ/, and /uː/–/ʊ/)? and (ii) how do the Greek speakers’ PSTM and nonverbal IQ capacities affect their discrimination abilities? The first question’s motivation stems from the limited research on the discrimination of English sound contrasts by Greek speakers. This research gap offers an opportunity to gain valuable insights into how an individual’s L1 influences the acquisition of L2 sounds, especially in the case of an understudied language like Greek. By exploring this, we can enhance our understanding of crosslinguistic phonology and, practically, help educators develop effective teaching strategies and provide language learners with tools to identify phonetic challenges. The second question arises from the noticeable scarcity of comprehensive research into the influence of PSTM and nonverbal IQ on L2 speech perception, especially in speakers with a Greek L1 background. This gap in knowledge hinders our ability to grasp why some language learners thrive in acquiring new phonological and phonetic elements while others encounter difficulties. Exploring the connection between PSTM, nonverbal IQ, and L2 speech perception can illuminate this phenomenon, contributing to a more profound comprehension of the cognitive underpinnings of language acquisition. This understanding can be a pivotal resource for educators seeking to tailor instruction to the cognitive profiles of individual learners, ultimately enhancing language learning outcomes.

Standard Modern Greek consists of a simple five-vowel system including vowels /i e a o u/ (see Georgiou & Themistocleous, 2021), while Standard Southern British English has a more extensive vowel system which includes tense and lax vowels, namely, /iː uː ɜː ɔː ɑː/ and /ɪ ʊ e æ ʌ ɒ/ respectively. This study concentrates on monophthongs as the few previous studies involving Greek speakers have focused on this type of L2 English vowels. As shown by previous research, most of these sounds are difficult for Greek speakers due to the size and complexity of the L2 vowel system and the differences between the L1 and the L2 vowels at the acousticophonetic level. For example, Lengeris (2009) studied the perception of English vowels by adult Greek learners of English, indicating sinlge-category assimilations for several English vowels. Specifically, English /iː/ and /ɪ/ were both assimilated to Greek category /i/, English /ε/ and /ɜː/ to Greek /e/, English /æ/ and /ʌ/ to Greek /a/, English /ɑː/, /ɒ/ and /ɔː/ to Greek /o/ and English /ʊ/ and /u:/ to Greek /u/. The discrimination scores showed that the most difficult contrast was /uː/–/ʊ/ followed by /ɑː/–/ʌ/, /ɔː/–/uː/, /æ/– /ʌ/, /iː/–/ɪ/, /ɔː/– /ɑː/, /ɒ/ –/ɔː/, /æ/–/ɜː/, and /ɪ/–/e/ in the /bVb/ context.

### Discrimination Predictions

**Fig. 1:**
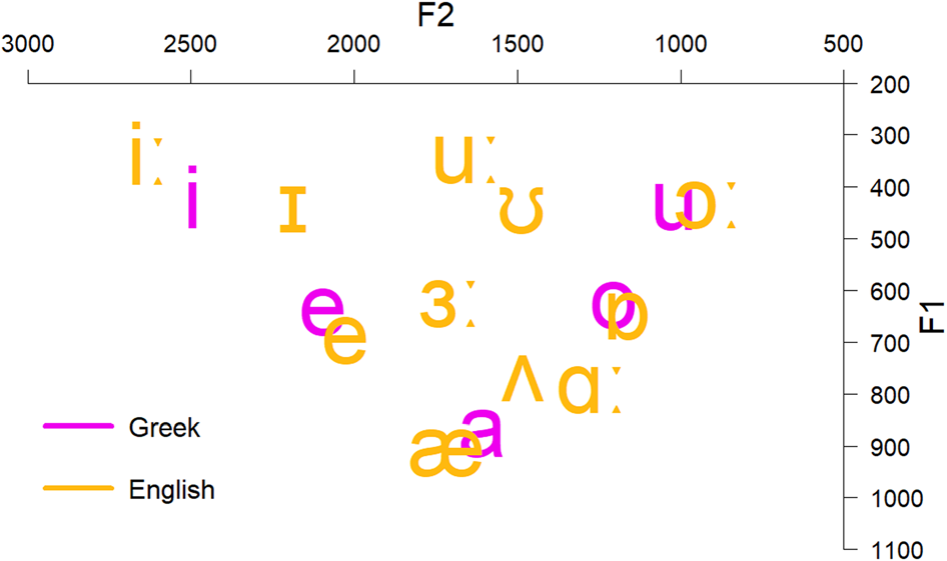
*F1* × *F2* (in Hz) of Greek and English vowels as produced by the respective native speakers

**Fig. 2:**
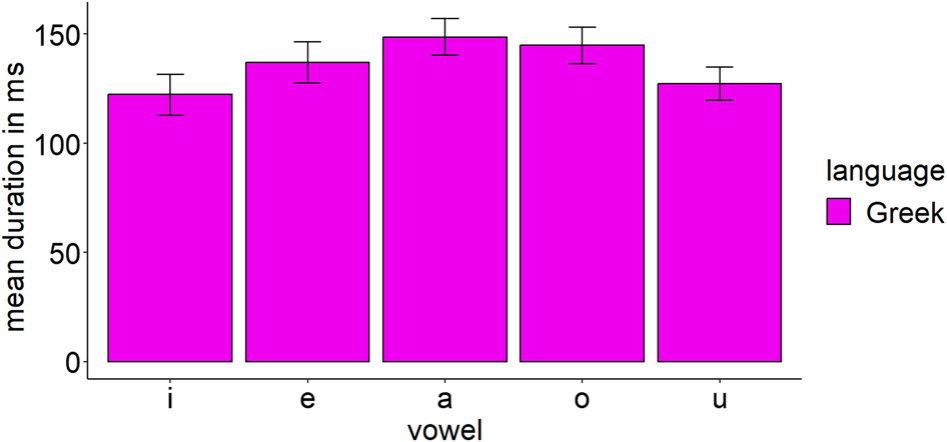
Duration of Greek vowels (in ms) as produced by Greek native speakers

**Fig. 3:**
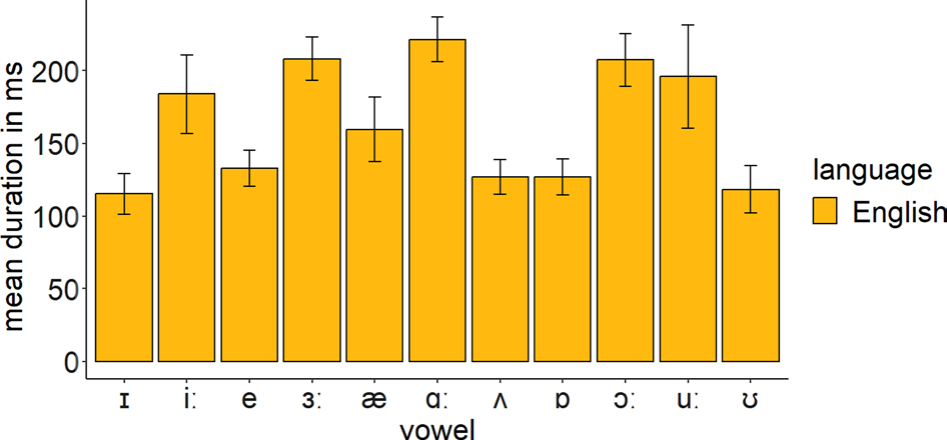
Duration of English vowels (in ms) as produced by English native speakers

We compared the acoustic characteristics of English and Greek vowels to form predictions about the discrimination of each L2 contrast. Eleven female adult Standard Modern Greek speakers were recorded producing the Greek vowels embedded in a /pVs/ context (5 vowels × 4 repetitions × 11 speakers = 220 productions). The words were part of the carrier phrase ‘Léne < target word > tóra’ (‘they say < target word > now’). In addition, 10 adult female Standard Southern British English speakers were recorded producing the 11 English vowels which were part of /hVd/ words (11 vowels × 2 repetitions × 10 speakers = 220 productions). The carrier phrase was ‘they say < target word > now’. All speakers were instructed to produce the stimuli as if speaking to a friend and were recorded at a 44.1 kHz sampling rate in quiet rooms. Only female speakers were used to eliminate the effect of sex on the productions. Figure 1 presents the *F1* × *F2* (measured in Hz) of Greek and English vowels as produced by native speakers of these languages. Figures 2 and 3 present the durations (measured in ms) and standard deviations (*SD*) of Greek and English vowels respectively as produced by native speakers of these languages.

According to the acoustic characteristics of English and Greek vowels, the following predictions can be formed about the discrimination of the target English contrasts by Greek speakers of English. These are based on the overlapping parameter threefold proposed by UPM:

- /iː/ –/ɪ/: English /iː/ is expected to be classified as Greek /i/ as it comprises its closer acoustic exemplar. English /ɪ/ is between Greek /i/ and /e/. However, its duration is close to the duration of English /iː/ and, therefore, it is expected to be mainly classified as Greek /i/. Thus, a completely overlapping contrast is expected, which will signal poor discrimination.
- /iː/–/e/: English /iː/ is expected to be classified as Greek /i/, while English /e/ will probably be classified to its closest exemplar, that is, Greek /e/. The contrast will be nonoverlapping, presenting excellent discrimination.
- /ɑː/–/ʌ/: English /ɑː/ lies between Greek /a/ and /o/, and thus it may overlap with both Greek vowels. However, its duration is closer to Greek /a/ and thus it may be classified in terms of that Greek vowel. English /ʌ/ might be classified as Greek /a/, which is its closest acoustic exemplar. The contrast is expected to be completely overlapping.
- /æ/–/ɑː/: English /æ/ is expected to be classified as Greek /a/, while /ɑː/ might be classified as Greek /a/. Complete overlap is expected.
- /ɔː/–/ɒ/: English /ɔː/ is close to Greek /u/, but its duration is closer to Greek /o/. Therefore, it might be an instance of Greek /o/ and /u/. English /ɒ/ is close to Greek /o/ and it might be classified in terms of that Greek category. The contrast is expected to be partially overlapping, exhibiting better discrimination than the completely overlapping contrasts but worse discrimination than the nonoverlapping contrast.
- /uː/–/ʊ/: English /uː/ is acoustically close to several Greek vowels. It is expected to be classified mostly as Greek /o/ or /u/ since it shares more acoustic properties with these vowels. English /ʊ/ is between Greek /o/ and /u/ but its duration is closer to the duration of Greek /u/. The contrast might be either completely or partially overlapping.

### Predictions About the Effect of PSTM and Nonverbal IQ

Although it is difficult to develop predictions for each English contrast, we hypothesize that both PSTM and nonverbal IQ will positively affect the discrimination of all or most contrasts since the majority of the previous studies demonstrated either an association of phonetic abilities with PSTM (e.g., Aliaga-Garcia et al., 2011; Safronova, 2016) or the positive role of nonverbal IQ in several linguistic skills (e.g., Łockiewicz et al., 2018; Woumans, 2019) including speech perception (e.g., Georgiou, 2023a).

### Hypotheses

Based on the above predictions, we aim to assess the following hypotheses:

#### H1

The control group (i.e., English speakers) will discriminate all but one L2 contrast (i.e., /iː/–/e/) with *higher* accuracy than the experimental group (i.e., Greek speakers). Also, evidence is expected to indicate *below* chance performance in the discrimination of these contrasts by the experimental group, with the control group anticipated to demostrate *above* chance performance.

#### H2

For the Greek speakers, the discrimination accuracy of English /iː/–/e/ will be *higher* compared to the accuracy of the other contrasts, and the discrimination accuracy of /ɔː/–/ɒ/ will be *higher* than the accuracy of the other contrasts except /iː/–/e/.

#### H3

The discrimination accuracy of the L2 contrasts will be *higher* for Greek speakers with high PSTM than for Greek speakers with low PSTM.

#### H4

The discrimination accuracy of the L2 contrasts will be *higher* for Greek speakers with high nonverbal IQ than for Greek speakers with low nonverbal IQ.

## Methodology

### Participants

Twenty monolingually-raised Standard Modern Greek speakers with an age range of 25–45 (*M*_*age*_ = 32.15, *SD* = 7.06) (*n*_females_ = 12) participated in the study. All participants originated from moderate-income families and reported that they were permanent residents of Athens, Greece. None of them had ever lived for a long time in an English-speaking country. According to their self-reports, they had knowledge of English at the B2/C1 levels and the mean of their understanding skills in English was 4.2/5 (*SD* = 0.68). The mean onset age of learning English was 7.75 years (*SD* = 1.73) and the daily use of English was 0.85 h (*SD* = 1.35) on average. The participants were divided into two groups according to their PSTM and IQ capacities (i.e., high/low PSTM, high/low IQ) after conducting a median split on the raw scores of the PSTM and nonverbal IQ tests. The control group consisted of 10 speakers of Standard Southern British English with an age range of 24–42, who permanently resided in the UK (*M*_age_ = 31.4, *SD* = 6.02) (*n*_females_ = 5). These participants had a moderate socioeconomic status. All participants had healthy vision and hearing.

### Stimuli

The stimuli consisted of the 11 English monophthongs embedded in monosyllabic /hVd/ words, which were part of the carrier phrase “They say < word > now”. These words were *hid, heed, head, herd, had, hard, hud, hod, hoard, who’d*, and *hood*, representing the vowels /ɪ iː e ɜː æ ɑː ʌ ɒ ɔː uː ʊ/ respectively. Two adult female Standard Southern British English native speakers were recorded at a 44.1 kHz sampling rate producing the carrier phrases. The speakers were instructed to produce the phrases as naturally as possible as if speaking to a friend. The stimuli were normalized for peak intensity in Praat (Boersma & Weenink, 2022).

### Procedure

#### Discrimination Test

All participants completed an AX discrimination test in Praat. The stimuli were grouped into six “different” pairs and six “same” pairs. The “different” pairs included the six English contrasts under investigation, that is, /iː/–/ɪ/, /iː/–/e/, /ɑː/–/ʌ/, /æ/–/ɑː/, /ɔː/–/ɒ/, and /uː/–/ʊ/. Each of the six “different” conditions contained eight repetitions of the contrastive vowels (4 AB and 4 BA types). Similarly, each of the six “same” conditions contained eight repetitions of the contrastive vowels (4 AA and 4 BB types). A total number of 96 items (6 contrasts × 2 conditions × 8 repetitions) were discriminated by each participant and all of them were presented in random order. The stimulus pairs always included recordings from different talkers. Participants were asked to sit in front of a PC, maintaining a consistent distance from it. They listened to the stimuli through a set of headphones connected to a PC and were asked to select whether the pair tokens were acoustically different or the same by clicking on the relevant script label. The interstimulus interval was 300 ms. During the experiment, the stimuli could not be repeated and no feedback was given on the participants’ responses. In addition, there was an optional five-minute break at the midpoint. Prior to the main experiment, participants completed a familiarization test with four items on the script to ensure that they understood the requirements of the test. The test lasted about 15–20 min for each participant.

#### PSTM Tests

Participants completed a digit span test to measure their PSTM capacities. The digit span test is typically used to measure PSTM together with other tests such as nonword repetition tests (Brunfaut & Révész, 2015; Perez, 2020). The former has been suggested to carry semantic information that can bias the performance of PSTM memory (Jacquemot & Scott, 2006), while the latter is not associated with such effects as it uses pseudowords. Nevertheless, even when employing nonwords (or pseudowords), it is important to recognize that these linguistic constructs are not entirely devoid of meaning and can still evoke semantic associations (Chuang et al., 2021). For this study, the digit span test was preferred because it has been widely used for the estimation of PSTM and it is relatively quick to administer, making it a convenient tool to assess PSTM capacity in a short amount of time. It is also easy to use and understand compared to nonword recognition/repetition tests. The test was completed on a PC in quiet rooms and required the subjects to type the sequence of digits they listened to from the PC loudspeakers; all of them used headphones. Participants took a seat in front of a PC monitor, maintaining an approximate distance of 1 m from it. Apart from listening to the digits, they could also see them on the monitor. The digits were spoken at a rate of approximately one digit per 1000 ms. Each sequence began with three digits and upon the successful record of the digits, the sequence increased in length by one digit. There were two different digit span subtests: the forward digit span (FDS) and the backward digit span (BDS) tests. In FDS, participants were asked to type the sequence of digits in the order originally presented, while in BDS, they typed the sequence of digits in the reverse order from the original presentation. For both subtests, upon an unsuccessful record of the correct digits by the participants, another chance was given to them and they continued from the sequence they left. Upon a second incorrect attempt, the test stopped. The score of the participants was the sum of the number of digits correctly recorded in FDS and BDS. Each individual took 10–15 min to complete the test.

#### Nonverbal IQ Tests

Participants’ nonverbal IQ was measured through Raven’s Standard Progressive Matrices test (Raven et al., 2000). The test was completed in quiet rooms individually, following the PSTM test. Participants were presented with a series of matrices or visual patterns, each of which had one element missing. They were anticipated to identify the missing element from the set of options provided; there were either six or eight options for each item. They did this by discerning the underlying rules, patterns, and relationships within the matrix and then circling the option that logically completed the pattern. A total number of 60 black and white items in five sets (e.g., A to E) of 12 items (e.g., A1–A12) were presented. The items within a set progressively became more complex, requiring a greater amount of cognitive capacity to encode and analyze. The test was completed within 40–45 min. The performance of the participants was measured using the raw scores.

### Statistical Analysis

*Bayesian* regression models were used to analyse the data. The statistical analysis took place in R (R Core Team, 2022) with the use of the *brms* package (Bürkner, 2017). There are many advantages to using a Bayesian model including its ability to deal with small samples of participants (see Escudero et al., 2020; Georgiou, 2023b; van de Schoot & Depaoli, 2014). Approximate leave-one-out (LOO) cross validation was conducted to select the best-fitting model by comparing models with different fixed and random factors. For the examination of the discrimination accuracy, the final model (Model 1) included *contrast* (six English vowel contrasts), *group* (experimental/control)*,* and *contrast* × *group* as fixed factors and *subjects*, and *contrast* and *group* within *subjects* as random factors. The final model for PSTM and nonverbal IQ (Model 2) included *contrast, group.PSTM* (high/low), *group.IQ* (high/low), and *contrast* × *group* as fixed factors and *subjects*, and *contrast* and *group.IQ* within *subjects* as random factors. Weakly informative priors were used, namely, student’s *t*-distribution with 3 degrees of freedom, a mean of 0, and a standard deviation of 2.5 (see Gelman et al., 2014, 2015). We employed the *Bernoulli* distribution since the dependent variable *response* was dichotomous (0 = incorrect, 1 = correct).

After fitting the models, we proceeded with hypothesis testing. The likelihood of the test hypothesis against its alternative was estimated through the consideration of *Evidence Ratio* (ER). According to the evidence categories for the Bayes Factor BF_12_ of Jeffreys (1961) as cited in Andraszewicz et al. (2015), an ER of > 100 represents extreme evidence, ER of 30–100 very strong evidence, ER of 10–30 strong evidence, ER of 3–10 moderate evidence, and ER of 1–3 anecdotal evidence *for* a given hypothesis. An ER of 1 shows no evidence at all. An ER of 1/3–1 represents anecdotal evidence, ER of 1/10–1/3 moderate evidence, ER of 1/30–1/10 strong evidence, ER of 1/100–1/30 very strong evidence, and ER of < 1/100 extreme evidence *against* a hypothesis. For practical reasons, we will consider an ER of > 10 and an ER of < 1/10 (or 0.1) as strong evidence for and strong evidence against a particular hypothesis respectively. Apart from ERs, we also report the posterior probabilities (PP).

## Results

### Discrimination

**Fig. 4:**
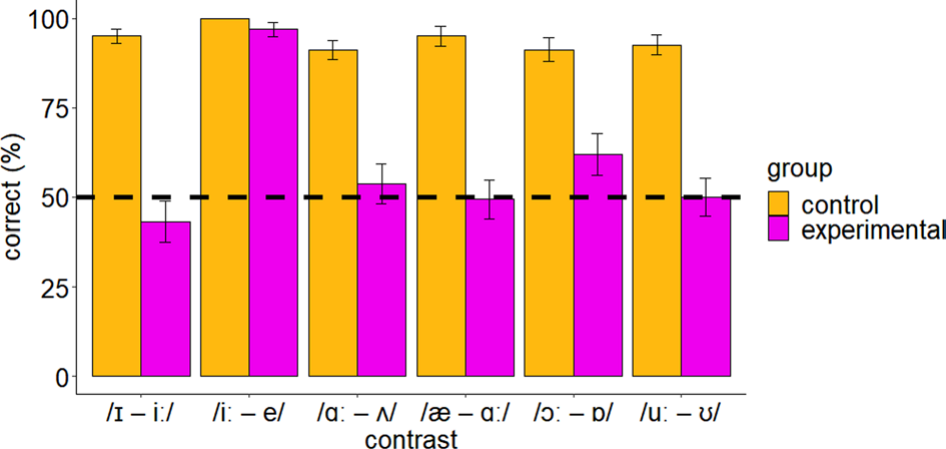
Discrimination accuracy of L2 English contrasts (percentage of correct responses) by the control (left) and the experimental (right) groups. The error bars show the *SE*s and the dashed line shows chance performance

The results of the discrimination test revealed that the experimental group exhibited poor discrimination against all but one L2 English contrast. The least discriminable contrast was /ɪ/–/iː/, followed by /æ/–/ɑː/, /uː/–/ʊ/, /ɑː/–/ʌ/, and /ɔː/–/ɒ/. The /iː/–/e/ contrast was discriminated with high accuracy. The control group discriminated all contrasts with higher accuracy than the experimental group. Figure 4 illustrates the accuracy percentages for the discrimination of the six English vowel contrasts by the control and experimental groups.

To investigate whether there is evidence for above chance discrimination of the L2 contrasts by Greek and English speakers, we used two Bayesian regression models. With respect to Greek speakers, there was strong evidence of above chance discrimination performance only for /iː/–/e/ and /ɔː/ – /ɒ/ (ER > 116.65, PP > 0.99). With respect to English speakers, there was strong evidence that all contrasts were discriminated above chance (ER > 499, PP = 1). Furthermore, for the examination of H1, we fitted a new Bayesian regression model, namely, Model 1, and conducted hypothesis testing. As shown in Table 1, there was strong evidence that all L2 contrasts were discriminated better by the control than the experimental group (ER > 104.26, PP > 0.99).

**Table 1:** Hypothesis testing results for ERs of the *contrast* × *group* interaction in Model 1

Hypothesis control > experimental	Estimate	Estimate Error	CI. Lower	CI. Upper	ER	PP
/ɪ/–/iː/	3.57	0.63	2.57	4.63	Inf	1.00
/iː/–/e/	3.10	1.66	0.79	6.07	104.26	0.99
/ɑː/–/ʌ/	2.46	0.56	1.57	3.43	3999.00	1.00
/æ/–/ɑː/	3.02	0.55	2.14	3.95	Inf	1.00
/ɔː/–/ɒ/	2.12	0.59	1.18	3.10	3999.00	1.00
/uː/–/ʊ/	2.84	0.60	1.88	3.88	Inf	1.00

The hypothesis tests whether the control group discriminated each L2 contrast more accurately than the experimental group

We also compared the discrimination accuracy of different L2 contrasts within the experimental group (H2) using further hypothesis testing. The results demonstrated strong evidence that English /iː/–/e/ was discriminated with higher accuracy than four contrasts (ER = inf, PP = 1) and that /ɪ/–/iː/ was discriminated with lower accuracy than /iː/–/e/ (ER = 0, PP = 0). Also, there was strong evidence that English /ɔː/–/ɒ/ was discriminated with higher accuracy than /uː/–/ʊ/ (ER = inf, PP = 1) and that /ɪ/–/iː/ and /æ/–/ɑː/ were discriminated worse than /ɔː/–/ɒ/ (ER < 0.04, PP < 0.04). In sum, the results show that /iː/–/e/ was discriminated more accurately than all the other contrasts, while /ɔː/–/ɒ/ was discriminated better than three contrasts and worse than one contrast, namely /iː/–/e/. The results of hypothesis testing for the comparison of the accuracy of different L2 contrasts as discriminated by the Greek speakers are shown in Table 2.

**Table 2:** Hypothesis testing results for the ERs of the *contrast* effect in Model 1

Hypothesis (contrasts)	Estimate	Estimate Error	CI. Lower	CI. Upper	ER	PP
1 > 2	−3.67	0.50	−4.51	−2.90	0.00	0.00
1 > 3	−0.50	0.33	−1.04	0.03	0.07	0.06
1 > 4	−0.31	0.33	−0.86	0.24	0.21	0.17
1 > 5	−0.89	0.34	−1.44	−0.36	0.00	0.00
1 > 6	−0.32	0.33	−0.87	0.24	0.20	0.16
2 > 3	3.17	0.50	2.39	4.03	Inf	1.00
2 > 4	3.37	0.50	2.59	4.23	Inf	1.00
2 > 5	2.78	0.50	2.00	3.63	Inf	1.00
2 > 6	3.36	0.50	2.58	4.22	Inf	1.00
3 > 4	0.20	0.34	−0.35	0.76	2.67	0.73
3 > 5	−0.39	0.34	−0.95	0.18	0.14	0.12
3 > 6	0.18	0.33	−0.35	0.73	2.51	0.71
4 > 5	−0.59	0.34	−1.13	−0.03	0.04	0.04
4 > 6	−0.01	0.33	−0.55	0.53	0.93	0.48
5 > 6	3.43	0.70	2.31	4.61	Inf	1.00
---						
1 = /ɪ/–/iː/, 2 = /iː/–/e/, 3 = /ɑː/–/ʌ/, 4 = /æ/–/ɑː/, 5 = /ɔː/–/ɒ/, 6 = /uː/–/ʊ/						

The hypothesis tests whether each L2 contrast was discriminated more accurately than any other by the Greek speakers

### PSTM

For purposes of testing whether the high PSTM group discriminated the L2 contrasts more accurately than the low PSTM group (H3), we fitted another Bayesian regression model, namely, Model 2. The results of hypothesis testing showed that there was no evidence for better discrimination of five L2 contrasts by the high PSTM group (ER < 5.07, PP < 0.84). The results of hypothesis testing are shown in Table 3.

**Table 3:** Hypothesis testing results for the *contrast* × *group.PSTM* interaction in Model 2

Hypothesis high > low	Estimate	Estimate Error	CI.Lower	CI.Upper	ER	PP
/ɪ/–/iː/	−0.35	0.57	−1.30	0.59	0.37	0.27
/iː/–/e/	−0.36	0.91	−1.86	1.11	0.52	0.34
/ɑː/–/ʌ/	−0.80	0.59	−1.76	0.13	0.09	0.08
/æ/–/ɑː/	−0.44	0.53	−1.30	0.42	0.27	0.21
/ɔː/–/ɒ/	−0.41	0.59	−1.40	0.53	0.32	0.24
/uː/–/ʊ/	0.56	0.58	−0.40	1.55	5.07	0.84

The hypothesis tests whether the high PSTM group discriminated each L2 contrast more accurately than the low PSTM group

### Nonverbal IQ

We conducted another hypothesis testing to estimate whether the high IQ group discriminated the L2 contrasts more accurately than the low IQ group (H4). We found strong evidence that /ɪ/–/iː/, /ɑː/–/ʌ/, /æ/–/ɑː/, and /ɔː/–/ɒ/ (ER > 11.86, PP > 0.92) were discriminated more accurately by the high IQ group compared to the low IQ group. Weaker evidence was found for two L2 contrasts (ER = 3.04–3.96, PP = 0.75–0.80). The results of hypothesis testing are shown in Table 4.

## Discussion

The present study investigated the discrimination of L2 English vowel contrasts by Greek speakers. It also examined the role of PSTM and nonverbal IQ in the discrimination of L2 contrasts. The participants were adult Greek speakers with experience in English. They completed three tests: an AX discrimination, a digit span, and an intelligence test in controlled environments. The results of all tests were analyzed with the use of Bayesian regression models. Apart from revealing the speech perception patterns of speakers with an underresearched L1, that is Greek, this study provided further evidence—in the relatively scarce literature—about the effect of PSTM and nonverbal IQ on L2 speech perception.

**Table 4:** Hypothesis testing results for the *contrast* × *group.IQ* interaction in Model 2

Hypothesis high > low	Estimate	Estimate Error	CI.Lower	CI.Upper	ER	PP
/ɪ/–/iː/	0.90	0.58	−0.06	1.85	14.81	0.94
/iː/–/e/	0.78	0.91	−0.68	2.33	3.96	0.80
/ɑː/–/ʌ/	0.81	0.57	−0.11	1.74	11.86	0.92
/æ/–/ɑː/	1.00	0.50	0.18	1.83	41.55	0.98
/ɔː/–/ɒ/	1.34	0.59	0.40	2.33	110.11	0.99
/uː/–/ʊ/	0.37	0.55	−0.56	1.26	3.04	0.75

The hypothesis tests whether the high IQ group discriminated each L2 contrast more accurately than the low IQ group

Greek speakers of L2 English have insufficient abilities to discriminate particular English vowel contrasts. This finding confirms H1 since we found strong evidence that the control group discriminated all L2 contrasts better than the experimental group. Also, we found strong evidence that all but two contrasts were discriminated by Greek speakers below chance, while English speakers discriminated all contrasts above chance. The confirmation of this hypothesis underlines the great effect of speakers’ L1 on L2 vowel discrimination since every L2 sound is filtered through the speakers’ native inventory. One explanation would be that the Greek vowel system is smaller and less complex than the English vowel system; therefore, two or more L2 sounds might have been accommodated to a single L1 category, creating perceptual challenges regarding the discrimination of these contrasts (for further evidence, see Fox et al., 1995; Iverson & Evans, 2007, 2009). An additional explanation would posit that the acousticophonetic differences between the vowels of the two languages interfered with the perception of the L2 sounds, leading to perceptual problems. The poor performance of the speakers can also be interpreted as a result of the infrequent use of English in their daily life and the limited exposure to L2 naturalistic stimuli, which is considered an important factor in improving learners’ L2 speech perception abilities (Georgiou, 2021b). Usually, active learners of an L2 (e.g., see Georgiou et al., 2020b) or individuals with naturalistic access to the L2 speech (e.g., see Georgiou, 2018) are characterized by better discrimination abilities due to more robust mental representations compared to nonactive learners and speakers who live in a context where the L2 is not dominant.

H2 was also confirmed. This hypothesis was based on previous evidence which suggested that the consideration of the acoustic properties of L1 and L2 vowels can predict to some extent the speakers’ L2 discrimination patterns. Indeed, our initial predictions developed on the basis of crosslinguistic acoustic similarity were verified by the discrimination results. Specifically, English /iː/–/e/, which was regarded as a nonoverlapping contrast, had higher discrimination accuracy than the other L2 contrasts, and English /ɔː/–/ɒ/, which was a partially overlapping contrast, had higher discrimination accuracy than the other L2 contrasts except /iː/–/e/. Therefore, crosslinguistic acoustic similarity can somehow be successful in predicting the discrimination accuracy of the L2 contrasts (see Alispahic et al., 2017; Elvin et al., 2014; Escudero et al., 2012; Georgiou, 2024). In addition, the overlapping parameters proposed by UPM can be a good metric for the estimation of L2 contrast discrimination accuracy (see Georgiou, 2022b).

Against our expectations, there was no evidence suggesting that speakers with high PSTM discriminate particular L2 English vowel contrasts more accurately compared to speakers with low PSTM. Therefore, H3 was rejected. This is inconsistent with several studies that have found positive effects of PSTM on L2 speech perception (e.g., Lengeris & Nicolaides, 2014; MacKay et al., 2001). However, it agrees with the findings of other studies that provide counterevidence. Safronova and Mora (2012) observed that Spanish/Catalan learners of English with a high PSTM capacity could not perceive the English contrast /ɪ/–/iː/ more accurately than learners with a low PSTM capacity. Also, Ghaffarvand Mokari and Werner (2019) reported that PSTM was not associated with gains of phonetic training in the discrimination of English vowel pairs. Our findings may have several explanations. For example, the outcomes may depend on the type of test used to assess speakers’ PSTM. In our study, we used a digit span test, while other studies reporting a positive effect of PSTM used nonword recognition or nonword repetition tasks. This result may have emerged from the fact that nonword tasks are more specific for the measurement of phonological processing and phonological information stored in PSTM compared to the digit span tests, which may additionally demand WM skills (especially in BDS) (Volpato, 2020). Notably, WM capacities were not found to be associated with L2 speech perception in some recent studies (e.g., Inceoglu, 2019), providing a justification for the nonexistent effect of PSTM on L2 speech perception in this study, which employed a digit span test. In addition, the results may suggest a complex interplay between PSTM and L2 sound perception. While PSTM is recognized as a contributing factor to L2 sound perception, the scope and character of its impact can vary significantly depending on factors such as the speakers’ L1 and L2, and linguistic materials such as contrasts under investigation in a given study. Therefore, the role of PSTM might not be universally consistent and may depend on the aforementioned factors. Another possible explanation for the results of this study is the role of perceived difficulty for each contrast. Considering that the processing of difficult contrasts requires larger cognitive demands (Ghaffarvand Mokari & Werner, 2019), a specific contrast which is perceived as difficult by an individual with high PSTM may be discriminated in the same manner by an individual with low PSTM who has perceived it as an easy contrast. Finally, other extralinguistic and extracognitive factors such as speakers’ daily fatigue or psychological stress may have also affected the results. This is because fatigue is associated with decreased motivation to apply effort to the task (Wang et al., 2018). Speakers’ psychological stress during the task (as the task was inspected live by the researchers) may have affected the participants’ performance as there is evidence that the presence of stress affects memory functions (Schoofs et al., 2009).

The findings demonstrated that there was overall evidence that speakers with high nonverbal IQ discriminated the L2 English contrasts more accurately than speakers with low nonverbal IQ. Strong evidence was found for four out of six L2 contrasts. Therefore, H4 can be accepted to a large extent, highlighting the positive role of nonverbal IQ in L2 speech perception. While these results diverge from earlier findings that showed no association between phonetic abilities and nonverbal IQ (e.g., Rota & Reiterer, 2009), they align with the results of Georgiou (2023a), providing further evidence supporting the connection between nonverbal IQ and phonetic (perceptual) abilities. Nonverbal IQ relates to several cognitive functions, which include perception, learning, and language abilities (Kiely, 2014). All these functions apply to speech perception, which requires listeners to extract acoustic information from the speech signal and organize speech sounds categorically in the mind. So, the advantage of high nonverbal IQ individuals may be attributed to their enhanced ability to learn and process information (see Stenberg, 1985), including phonological and phonetic relationships. Another explanation is that speakers with high nonverbal IQ have increased abilities in controlling their attention (Sweller, 1988). This means that they can potentially attune to the L2 sound patterns more easily compared to speakers with low nonverbal IQ. For all these reasons, the link between nonverbal IQ and speech perception seems reasonable and speakers with high nonverbal IQ are likely to perform better in speech perception tasks than speakers with low nonverbal IQ. We propose that the effect of nonverbal IQ on L2 phonological discrimination is contrast-specific since not all contrasts were found to be affected to the same extent. At this stage, we are not able to explain why nonverbal IQ did not affect the discrimination of particular contrasts. Perhaps, this is related to the level of the perceived difficulty of each contrast, just like in the case of PSTM, which may prevent speakers with high nonverbal IQ from perceiving acoustic differences better than speakers with low nonverbal IQ.

## Conclusions

Our findings suggest that Greek speakers demonstrate poor performance in the discrimination of the majority of L2 English contrasts. In addition, PSTM did not have any effect on L2 speech perception, while there was an effect of nonverbal IQ on most contrasts. Although we attempted to control several factors such as L2 proficiency, L2 use, age of L2 learning onset etc. other factors such as speakers’ motivation, attention control, phonetic aptitude, etc. were not considered for practical reasons; these unexamined factors may have affected the results, underscoring the need for their comprehensive examination in subsequent studies. Also, we used a single tool to measure PSTM and nonverbal IQ capacities. While these measures provided valuable insights into the association between cognitive functions and L2 speech perception, incorporating multiple tools and assessments would offer a more comprehensive understanding of this relationship. For example, a future study can include additional measures of PSTM and nonverbal IQ using a variety of tools such as nonword repetition and nonword recognition tasks and the WASI test respectively. Finally, considering the varying effects of cognitive measures on each L2 contrast, future studies may explain why particular L2 contrasts are affected differently by measures of cognitive functions.

## Data Availability

Not applicable.
